# Supplementary material for: Predictors for liver fibrosis in non-alcoholic patients with psoriatic diseases: A multicenter cross sectional-study
Source: PLoS One. 2024 Apr 16;19(4):e0290632. doi: 10.1371/journal.pone.0290632 (PMC11020486; doi:10.1371/journal.pone.0290632)
Supplement: Attachment — Submitted filename: rebuttal letter psoriasis 080723.docx [file pone.0290632.s003.docx]

Dear reviewers,

We appreciate very much you agreed to evaluate our study, we already made the changes you suggested. The modifications are detailed below:

1. - Did you test the quantitative variables regarding their normality of distribution? Please mention the name of the test in the statistical analysis section.

R: We added in the data analysis section the following paragraph: “We used the Kolmogorov-Smirnov test to test the normality of the data, since nonparametric distribution exists, we reported continuous data with median and interquartile range”.

2. Please also mention the tests that were used for evaluating the variables with and without a normal distribution in the statistical analysis section (e.g. t-test and its nonparametric equivalent).

R: We added in the data analysis section the following: “We test the difference between patients with fibrosis and without fibrosis using Chi-squares for proportions and performed Mann-Whitney U test to compare continuous outcomes”.

3. - Please make sure to use the mean +/- standard deviation for the variables with a normal distribution and the median [IQR] for the variables without a normal distribution (in the result's text and tables).

R: We changed the results that were reported with mean and SD with median an IQR in both the result section and the table.

4. Please make sure to use parametric tests to evaluate the variables with normal distribution and non-parametric tests to evaluate the variables without a normal distribution (in the result's text and tables).

R: We changed the results that were reported with mean and SD with median an IQR in both the result section and the table.

5. The style/formatting of tables 2 and 3 need to be changed. Please change them to a simple plain style.

R: We changed the style of tables 2 and 3.

6. The discussion part needs to be improved. Please use some recent studies to expand the discussion.

R: We changed some sections on the discussion.

Norberto Chavez-Tapia, M.D., PhD
